# Scoping the Scene: What Do Nurses, Midwives, and Allied Health Professionals Need and Want to Know About Genomics?

**DOI:** 10.3389/fgene.2019.01066

**Published:** 2019-11-12

**Authors:** Mona Saleh, Romy Kerr, Kate Dunlop

**Affiliations:** ^1^Centre for Genetics Education, New South Wales Health, Sydney, NSW, Australia; ^2^New Zealand Genetic Health Service (Northern), Auckland, New Zealand

**Keywords:** genetics, genomics, allied health, education, nurse, Australia, midwife, knowledge

## Abstract

**Introduction:** Rapid changes in genomic technology are transforming healthcare delivery. Although it has been well established that many health professionals lack the adequate knowledge, skills, and confidence to adapt to these changes, the specific educational needs of Australian allied health professionals, nurses, and midwives are not well understood. This diverse group of health professionals is primarily involved in the management of symptoms and psychosocial care of patients with genetic conditions, rather than risk assessment and diagnosis. The relevance of genetics and genomics to their clinical practice may therefore differ from medical practitioners and specialists.

**Materials and Methods:** This paper reports on a study undertaken to identify the perceived genetic knowledge and education needs for this group of health professionals. Allied health professionals, nurses, and midwives were recruited from throughout New South Wales (NSW) and invited to participate in semi-structured telephone or face to face interviews.

**Results:** A total of 24 geographically and professionally diverse individuals (14 allied health, 6 nurses, and 4 midwives) were interviewed. Interview recordings were transcribed and using thematic qualitative analysis recurring themes were identified. The results show that this is a diverse group that is keen to know more about genomics and genetic services but unsure of reliable sources.

**Discussion:** The need for a generic update from a trustworthy source was identified and suggested topics to be covered included genetic fundamentals, recognizing common genetic conditions, and psychosocial/ethical aspects of genetics/testing including informed consent. In addition, the challenge of incorporating education into highly clinical roles was identified as a key barrier and having a readily accessible, accredited learning resource would help overcome this. Findings from this study are informing the development of a targeted, interactive e-learning resource for allied health professionals, nurses, and midwives.

## Introduction

The advances in genomic technology and the advent of genomic medicine are changing healthcare delivery and the educational requirements of health professionals. Where previously genetic testing was most often limited to single gene tests for conditions with a clear phenotype (Bowdin et al., 2016), non-targeted, high-resolution next-generation sequencing technologies are now able to detect disease-causing changes in uncharacterized genes; identify an increased risk for complex conditions; predict disease development in the absence of symptoms; determine individual drug metabolism and efficacy; and identify personalized targeted therapy approaches (Mattick et al., 2014). Clinical genomics is moving beyond clinical genetics services to care management and treatment decisions in general medicine. This increase in utility and accessibility of genomic technology has resulted in an increased use of genomics by non-genetic healthcare providers and a change in their required knowledge and skillset (Campion et al., 2019).

Allied health professionals (university qualified health professionals with a non-medical, dental, or nursing qualification such as physiotherapists and pharmacists), nurses, and midwives are a diverse group of health professionals and as such their use of relevant genetic knowledge and skills varies. Nonetheless, many will be involved in both independent therapies or multidisciplinary work where they will encounter genetics in their clinical practice (Calzone et al., 2010; Crane et al., 2012). A survey of 3,600 American allied health professionals found that 70% of respondents reported discussing the genetic basis of health concerns with their clients and 30% reported providing counseling for genetic concerns (Lapham et al., 2000). Moreover, Barnoy et al. (2010) demonstrated that patients regarded advice about genetic testing from expert nurses and expert physicians as equally valuable, indicating a high level of trust between patients and nurses and the value of nurses with good genetics knowledge in healthcare.

The effective implementation of genomic medicine in the health system relies upon non-genetic health professionals remaining abreast with current genomic knowledge and confidently applying genetic skills in their practice. This requires maintaining a good understanding of basic genetic concepts; the current capabilities and limitations of genomic technology; the social, ethical, and psychological implications of genetic testing; the relevance of genomic medicine to clinical practice; and an awareness of available services and the confident use of skills such as family history taking and result interpretation (Bowdin et al., 2016; Tonkin et al., 2018;Wynn et al., 2018). In Australia, the National Health Genomics Policy Framework 2018–2021 focuses on integrating genomics into the healthcare sector through five main strategies, including ensuring a healthcare workforce that is literate in genomics as a priority (Council, 2017).

Despite this evidence to support the need for allied health professionals, nurses, and midwives to be equipped with genetics and genomics knowledge and skills, fewer than 30% of allied health professionals report a high level of confidence in carrying out tasks relating to genetics (Lapham et al., 2000). Over 80% of registered nurses and midwives who participated in a 2016 Australian study indicated the perception that their knowledge of genetics was poor to average (Wright et al., 2019). A systematic review of published studies reporting nurses’ competence in genetics found that nurses in the United Kingdom, Europe, and the United States of America lacked the required genetics knowledge and skill to meet their national core competencies (Godino and Skirton, 2012).

Much of the existing research focus has been on the educational needs of doctors (Lapham et al., 2000; Houwink et al., 2011; Nair et al., 2018; Rubanovich et al., 2018). Some research has focused on the educational needs of nurses and midwives particularly around confidence levels (Maradiegue et al., 2008; Calzone et al., 2010; Crane et al., 2012; Godino and Skirton, 2012; Skirton et al., 2012; Calzone et al., 2013; Wright et al., 2019), with limited understanding of the educational needs of allied health professionals (Neils-Strunjas et al., 2004; Christianson et al., 2005; Zant et al., 2015; Brown et al., 2019). Furthermore, only more recently has research focused specifically on the genomics education needs of non-genetic health professionals. There remains, therefore, a gap in understanding how allied health professionals, nurses, and midwives perceive the impact of genomic medicine on their clinical practice or what their educational needs are.

Importantly, this is a clear gap for those health professionals practicing in Australia. Much of the research addressing their genetic and genomic educational needs originates from the United Kingdom or the United States of America and Canada. The Australian Genomics Health Alliance has undertaken comprehensive needs assessment of medical specialists and general practitioners in genomics education but has yet to target this group (see https://www.australiangenomics.org.au/resources/publications/reports/).

This study aims to explore this gap in the understanding of the genomic educational needs of allied health professionals, nurses, and midwives working in Australia through a qualitative exploration of allied health professionals’, nurses’, and midwives’ perceptions of their knowledge of genetics and genomics and its relevance for their clinical practice.

The findings of this study will be used to inform an educational strategy and resources for allied health professionals, nurses, and midwives aimed at addressing the identified educational needs.

## Materials and Methods

### Participants

The study was approved by the Human Research Ethics Committee Review Board of Northern Sydney Local Health District. Allied health practitioners, nurses, and midwives in New South Wales (NSW), Australia were recruited using a number of targeted strategies. A letter of invitation and information flyer was sent to previous professionals in this group who had contacted The Centre for Genetics Education (CGE) for professional development in genomics over the past 2 years and also to relevant health service managers and department heads throughout NSW. The net was cast as widely as possible in order to recruit from a broad geographical area and a range of clinical specialties. Recruitment materials were sent to the NSW Ministry of Health Chief Nursing and Midwifery Officer and Committee and the Chief Allied Health Officer and Committee, as well as through local health networks including the NSW employee mailing lists through appropriate channels and with appropriate permissions. Included in the invitation to participate was a request to share the project invitation and flyer to colleagues. The promotional flyer was also placed in local health district newsletters and on staff notice boards. Flyers were also distributed to NSW clinical genetics services and genetic outreach genetic counselors (see www.genetics.edu.au).

Contact details for the researchers were included on the invitation to participate and the promotional flyer. Those who wished to participate were required to contact the researchers to indicate their interest. Interested health professionals were then sent a recruitment pack containing a participant information statement, consent form, and reply-paid envelope (if necessary). Interested participants who had not returned their consent forms 2 weeks after the initial contact were followed up by phone or email to remind them of the study and to request they return their signed consent forms if they still wished to participate. Health professionals who consented to participate were contacted to arrange a mutually agreeable time and location for a telephone or face-to-face interview.

Recruitment continued until data saturation was reached.

### Instrumentation

An interview guide adapted in part from Reed et al. (2015) was developed and conducted with participants either face to face or over the telephone. It consisted of demographic questions followed by semi-structured and open-ended questions about participants’ understanding and training in genetics and genomics; their experience of genetics in their practice; their confidence using genetic knowledge and skills; and their perceived genetic and genomic educational needs. Probes were used to encourage thorough exploration of the participant’s experiences and opinions. The interviews were carried out by either MS or RK (supervised by MS). Recruitment was ceased once there was no new information or themes being observed in the interviews. Interviews were digitally recorded and transcribed verbatim.

### Data Analysis

Using QSR International’s NVivo 11 qualitative data analysis software and using thematic qualitative analysis, index themes and categories were identified within the textual data. Categories were verified by at least two of the authors to maintain inter-rater reliability and increase validity (Miles and Huberman, 1994; Krueger and Casey, 2000; Pope et al., 2000). All the data relevant to each category were then identified, contextually defined (by referring back to the audio and/or transcripts), and coded manually. Themes recognized through this process were documented including illustrative verbatim comments from participants. RK identified the initial themes and categories and coded all transcripts. Five of these were then coded independently, using the developed categories, by MS. There was 100% consensus between both coders with regards to the main themes identified. Where small discrepancies occurred with respect to specific categories, discussions were held until consensus was reached.

## Results

### Participant Characteristics

A total of 24 interviews were carried out with participant characteristics shown in **Table 1**. The majority of participants were female and the mean age of participants was 48 years with an average years of practice being 18.7 years.

**Table 1:** Participant demographics.

Profession	Male (n)	Female (n)
**Allied health**		**14**
Occupational therapist	1	3
Dietician		3
Speech pathologist	1	1
Physiotherapist	1	2
Pharmacist		1
Social worker		1
**Nurse**		**6**
**Midwife**		**4**

Main group totals are in bold.

### Qualitative Findings

All participants acknowledged the importance of up-skilling in genomics. The extent and focus of these skills, however, and where to find appropriate education were not clear to most of those interviewed. The challenge in recognizing the relevance of genomics information was also reflected in many interviews with one participant summing this up by stating:

“I think that you don’t know what you don’t know until someone tells you. It’s [genetics] often discussed at a higher level rather than actually explaining things properly so people don’t recognize that it would be of value to your work”—Nurse (P05)

Overall, four distinct themes arose from the qualitative data: 1) existing genomics knowledge or exposure in practice; 2) relevance of genomic knowledge/skills to profession; 3) education and other challenges of incorporating genomics into practice; and 4) potential genomics topics to be incorporated into training.

Below is a summary of these themes and subcategories with evidence from transcripts to illustrate the issue.

#### Existing Genomics Knowledge or Exposure in Practice

The majority of participants felt that their graduate qualifications contained little if any genetics. If there was some genetics, it was very basic and therefore any relevant and applicable genomic education was sought out as an additional qualification or individual training.

##### During Undergraduate Degree

“In terms of training, basically no. I have a Bachelor of Applied Science in Physiotherapy and naturally there’s physiology, DNA and a certain amount of understanding of genetics from that, but it’s basic.”—Physiotherapist (P01)

“We did touch on it but it wasn’t as deep as what I expected it to be and I just feel the average nurse would like to know more because you open up a Pandora’s Box we get told how important it is but unless you do a degree in medicine, I suppose you wouldn’t know.”—Nurse (P05)

##### On-the-Job Training

Recognizing an interest and need for improved genomic knowledge, some participants revealed how and where they had sought out further education either formally or through interactions with peers.

“Last year I organized for a geneticist and genetic counselor to come and speak to our team and give us an update. It’s tricky to organize with everyone’s schedules but it’s worth it.”—Occupational Therapist (P18)

“I’ve just learnt through osmosis. It’s not a taught thing, just more working with the consultants and watching them take histories and things.”—Nurse (P16)

There were no participants who had undertaken any formal genetics training.

##### Interactions With Genetic Professionals and Services

Participants had variable interactions with genetic services. They felt that doctors, rather than allied health practitioners, nurses, and midwives, would be more likely to have direct interactions with genetic services. Others, however, who worked closely with or were linked to a genetic service appeared to possess some confidence/insight into genomic knowledge and referral pathways for patients. While this was a positive finding, unfortunately, there were others with limited contact and had little awareness of what genetic services were available, what they offered, and how to contact them.

“Yes definitely yes I would just ring the [Geneticist] on call they are very approachable. Often they can answer queries on the phone but if not they will address the issue another day or they will come and see the patient. They’re very good.”—Midwife (P09)

“I wouldn’t know where to refer them but I think I would probably get on the internet and search through a website and possibly do a preliminary phone call to make sure that was the correct service for that person to be referred to and then refer them on to that.”—Social Worker (P04)

#### Relevance of Genomic Knowledge/Skills to Profession

Midwives and nurses were more likely to feel that genomics and rapidly changing screening and testing options meant that they needed to keep abreast of current practices. They tended to rely on their professional societies and colleges to ensure they remained up to date. Attending relevant conferences or individual reading was mentioned as a way of staying informed. Some even learnt from their patients.

“A lot of women have the nuchal translucency and the [brand name] test and something else too, something ‘NIP’… I’m not sure because that’s all moved very quickly and because we don’t deal with these things it’s the women telling me what they’ve had rather than me understanding what they’ve had as such.”—Midwife (P22)

“We have in-services occasionally from our genetics team here, but I’ve had no training.”—Midwife (P09)

##### Family Health History as a Practice Tool

With regards to taking a family health history (FHH) and its relevance to their practice, once again it was nurses and midwives who expressed their opinion that this was relevant and in fact some responses showed a good understanding of the principal of taking a family health history. Others revealed a lack of technical understanding, feeling only maternal history was relevant.

“Yes, we take an obstetric and health history and family history, medical history; Gynecological history; Consanguinity; Standard questions.”—Midwife (P08)

“Family health history yes we do basically looking at maternal family history we do ask about deafness, blindness any Down syndrome and any genetical or hereditary abnormalities in the family. Just maternal only.”—Midwife (P09)

Responses from allied health professionals showed that they generally played a symptom focused role with each individual patient and therefore family health history was not seen as a priority. For specific symptomatic issues, however, family history was seen as relevant.

“I work from very much a functional point of view so if there’s a functional problem then I deal with that. I mean I could get carried away with the genetics and things like that but don’t, but sometimes it would be interesting to have a bit of an understanding of that.”—Occupational Therapist (P18)

“No we don’t do that. I mean I take a general family history especially with stuttering. I would just ask more general questions does anyone in the family have any speech language or learning delay or issues.”—Speech Pathologist (P11)

#### Education and Other Challenges of Incorporating Genomics Into Practice

Participants expressed the difficulty of incorporating continuing education into their work day. A lack of time as well as difficulty finding relevant and appropriate education were given as the most common reason for this. Having education provided and supported by the employer and also earning professional/ongoing education points for the professional were seen as the best possible way to incorporate genomic education. Also having targeted learning for specific health areas was seen as something more attractive, particularly to allied health specialists.

“I’m dubious about a lot of people going around with their shingles (office/business) providing professional development, I’m aware there are a lot of fad treatments out there and that sort of thing and I think that I would probably look at ones that has been around for a little bit longer and have research to back them up.”—Speech Pathologist (P11)

“I think pharmacists probably will only be particularly interested in medication effects so you’d have to tailor it that way for it to be relevant.”—Pharmacist (P17)

“Whether it’s about raising awareness at the management level that can then be filtered down through allied health departments, greater availability of Continuing Professional Development (CPD) events, they [employers] might support that, and CPD events that are perhaps targeted to allied health so that we see them advertized and think oh yeah it probably is worth my while going to that, whereas at the moment if I see a genetics talk advertized I would be likely to just dismiss it as something that’s more for the doctors than for me.”—Occupational Therapist (P20)

#### Potential Genomics Topics That Should Be Incorporated Into Training

Participants were given an opportunity to express their perceived topics of interest and those that should be made a priority in any future genomics education packages for allied health practitioners, nurses, and midwives. Participants were prompted by being asked, “What do you feel the genetic and genomic educational needs are for your profession and what suggestions do you have for incorporating genetics and genomics education into your training/professional development?”

These are listed in **Table 2** with genetic fundamentals and genetic conditions specific to professional roles mentioned as most relevant.

**Table 2:** Genetic and genomic topics preferred by participants.

Topic	Number of participants requesting topic
Genetic fundamentals	18
Genetic conditions specific to practice/role	*15*
Understanding genetic testing	*11*
When and how to refer to genetic services	*9*
Psychosocial implications	*8*
Current evidence and research	*8*
Ethical implications	*7*
Genomics	6
Understanding professional roles within genetics	5

## Discussion

This qualitative study is one of the first to explore the educational needs of allied health professionals, nurses, and midwives in Australia, and includes their experience of genetics and genomics to inform education. Most other studies for this group of health professionals have focused on confidence and relevance of genomics for their practice and the level of genomic literacy. Wright et al. (2019) reported a high perceived relevance or importance of genomics to practice among Australian nurses and midwives but a low level of genomics knowledge. A recent US study of audiologists (2019) and speech pathologists reported low confidence in their ability to implement principles of genetics, but over two-thirds agreed genetics was relevant for their field (Peter et al., 2019).

We found overall that Australian allied health professionals, nurses, and midwives are aware of the importance of up-skilling in genomics but remain unclear about how it applies to their practice. We did not find that genomics was necessarily seen as relevant to their practice and that some felt genomics primarily belonged with the medical profession. Genetics and genomics have not traditionally been central to the practice of most allied health professionals and nurses. Midwives, due to awareness of prenatal testing, reported far greater exposure and were the most familiar with genetic services and understanding and recording of Family Health History. Despite this, genomic literacy has been reported in Australia as generally low in midwives (Wright et al., 2019). Allied health professionals in our study felt that their limited exposure to genomics may be related to their specific roles, which often focus on functional problems rather than diagnosis. Zant et al. reported that physical therapist educators didn’t recognize the need for education due to the lack of perceived clinical applicability despite practicing physical therapists in this US study agreeing to the importance of increased genetic-related knowledge.

Participants in our study felt that reliable and relevant genomic education was not visible, and there was a lack of awareness about the role and existence of genetic services. All groups reported challenges in incorporating continuing education in their practice and highlighted the value of having education provided and supported by management and authority. Similarly, Campion et al. (2019) recommend the importance of service and educational activities of health professionals to be valued by genetics chairs and chiefs in the US. Genomics has a low profile in nursing in Australia at present (Wright et al., 2019). A mapping exercise of genomics education and training by the Australian Genomics Health Alliance Program 4 in 2018 did not identify any substantive Australian education programs for allied health professionals, nurses, and midwives except in the area of nutrigenomics for dieticians (McClaren et al., 2018). Internationally there have been significant efforts to provide accessible genomics education in particular the Health Education England’s Genomics Education Programme Nursing and Midwifery Transformational Strategy, which includes postgraduate training programs and genomic competencies for nurses (Tonkin et al., 2018). Also, in the United States and Canada, a number of organizations provide accessible genomics continuing education and resources such as the NIH National Human Genome Research Institute, Jackson Laboratory, and the American Medical Association (Campion et al., 2019). However, the impact on knowledge and practice of nurses, midwives, and nursing and allied health professionals has not been reported.

Our findings indicate that genetics fundamentals as a topic were the highest priority for this group when asked about their topics of interest, followed closely by genetic conditions and genetic testing. Selecting such broad topics may reflect their lack of confidence in knowledge and the lack of genetics and genomics in their undergraduate training. While this provides a good starting point for education resource development, it is interesting that participants acknowledged and volunteered the need to learn this content, but did not demonstrate interest to seek out opportunities independently. Some allied health professionals requested targeted education reporting that generic genomics education may not necessarily be seen as relevant or a priority learning area in the clinical setting. Stevens et al. found that most nurses were aware of the importance of genetics in relation to a specific disease highlighting this need for a connection to practice. While up-skilling is seen as important, it does not necessarily equate to interest (Wright et al., 2019). Therefore overcoming this mismatch may be complex and require in these early efforts well-targeted programs to reach and engage particular groups.

## Limitations

The participants were recruited from NSW only, were self-selected, and just over a quarter had a previous connection with the researchers, which may have led to a more informed group of participants than the workforce generally. In addition, the recruitment invitations included the words “genetics” and “genomics,” which in retrospect may have deterred those with no prior knowledge. However, due to the wide-reaching recruitment process, we were able to recruit a cross section of health professionals to represent the target group. Genomics knowledge was not assessed and therefore the study has no measure of what participants understood to be a satisfactory level of understanding. A limitation of the study was that allied health professionals have distinctly different roles from nurse and midwives but also among the different specialties, so it may be hard to generalize detailed findings for allied health professionals. However, themes were easy to identify and were consistent among researchers, and there was general consensus among all participants for the main themes.

## Future Directions

To adequately up-skill a workforce who lack understanding of the fundamentals of genomics and who struggle to see the relevance to their own clinical practice demands much more than incidental on-the-job training. A comprehensive and concerted approach to engaging this group in education that is targeted and relevant is required along with ongoing conversations among educators and healthcare managers to raise the profile of the importance of this education. Further research could explore the needs of specific groups of allied health professionals in genomics education and the impact of genomics education programs on knowledge and practice of nurses, midwives, and allied health professionals to further inform educational approaches.

In conclusion, our results suggest that allied health professionals, nurses, and midwives are aware of the importance of up-skilling in genomics and the need for educational resources particularly in the fundamentals of genomics. However, with few Australian education programs available, the inability to find relevance in genomics and the challenges in accessing education, nurses, midwives, and allied health professionals may fail to engage. Findings from this study will inform the development of an online genomics module and resources to be located on a state-wide education site that can be used as the foundation for targeted programs. Developing a workforce that is literate in genomics will require the development of accessible and innovative targeted education programs with support at policy and clinical level to reach and engage this group.

## Data Availability Statement

The datasets generated for this study are available on request to the corresponding author.

## Ethics Statement

The studies involving human participants were reviewed and approved by Northern Sydney Local Health District HREC. The patients/participants provided their written informed consent to participate in this study.

## Author Contributions

MS and RK designed the study with advice from KD. MS and RK carried out the interviews and analyzed the data. Writing of this manuscript was managed by MS. However, there was input from all authors.

## Conflict of Interest

The authors declare that the research was conducted in the absence of any commercial or financial relationships that could be construed as a potential conflict of interest.

## References

[B1] Barnoy S. Levy O. Bar-Tal Y. (2010). Nurse or physician: whose recommendation influences the decision to take genetic tests more? J. Adv. Nurs. 66 (4), 806–813. 10.1111/j.1365-2648.2009.05239.x 20423368

[B2] Bowdin S. Gilbert A. Bedoukian E. Carew C. Adam M. P. Belmont J. (2016). Recommendations for the integration of genomics into clinical practice. Genet. Med. 18(11), 1075–1084. 10.1038/gim.2016.17 27171546 PMC5557020

[B3] Brown H. D. Boonme K. Imrhan V. Juma S. Vijayagopal P. Prasad C. (2019). Should ‘Omics’ education be a part of allied health profession curricula? Genomics. 10.1016/j.ygeno.2019.01.009 30735794

[B4] Calzone K. A. Cashion A. Feetham S. Jenkins J. Prows C. A. Williams J. K. (2010). Nurses transforming health care using genetics and genomics. Nurs. Outlook 58 (1), 26–35. (In Press). 10.1016/j.outlook.2009.05.001 20113752 PMC2835985

[B5] Calzone K. A. Jenkins J. Culp S. Bonham V. L. Jr. Badzek L. (2013). National nursing workforce survey of nursing attitudes, knowledge and practice in genomics. Per. Med. 10 (7). 719–728 10.2217/pme.13.64 PMC3866033 24363765

[B6] Campion M. Goldgar C. Hopkin R. J. Prows C. A. Dasgupta S. (2019). Genomic education for the next generation of health-care providers. Genet. Med. 1–9 10.1038/s41436-019-0548-4 31110330

[B7] Christianson C. A. McWalter K. M. Warren N. S. (2005). Assessment of Allied Health Graduates’ Preparation to Integrate Genetic Knowledge and Skills into Clinical Practice. J. Allied Health 34 (3), 138–144. 16252676

[B8] Council. A. H. M. A. (2017). National health genomics policy framework 2018–2021. Health, Do, editor. Canberra: Australian Capital Territory.

[B9] Crane M. J. Quinn Griffin M. T. Andrews C. M. Fitzpatrick J. J. (2012). The Level of Importance and Level of Confidence that Midwives in the United States Attach to Using Genetics in Practice. J. Midwifery Women’s Health 57 (2), 114–119. 10.1111/j.1542-2011.2011.00132.x 22432481

[B10] Godino L. Skirton H. (2012). A systematic review of nurses’ knowledge of genetics. J. Nurs. Educ. Pract. 2 (3), 173–184 10.5430/jnep.v2n3p173

[B11] Houwink E. J. van Luijk S. J. Henneman L. van der Vleuten C. Dinant G. J. Cornel M. C. (2011). Genetic educational needs and the role of genetics in primary care: a focus group study with multiple perspectives. BMC Family Pract. 12 (1), 5. 10.1186/1471-2296-12-5 PMC3053218 21329524

[B12] Krueger R. A. Casey M. A. (2000). Focus Groups: A Practical Guide for Applied Research. California: Sage.

[B13] Lapham E. V. Kozma C. Weiss J. O. Benkendorf J. L. Wilson M. A. (2000). The gap between practice and genetics education of health professionals: HuGEM survey results. Genet. Med. 2 (4), 226–231. 10.1097/00125817-200007000-00005 11252707

[B14] Maradiegue A. Jasperson K. Edwards Q. T. Lowstuter K. Weitzel J. (2008). Scoping the family history: assessment of Lynch syndrome (hereditary nonpolyposis colorectal cancer) in primary care settings–a primer for nurse practitioners. J Am. Acad. Nurse Pract. 20 (2), 76–84. 10.1111/j.1745-7599.2007.00282.x 18271762

[B15] Mattick J. S. Dziadek M. A. Terrill B. N. Kaplan W. Spigelman A. D. Bowling F. G. (2014). The impact of genomics on the future of medicine and health. Med. J. Aust. 201 (1), 17–20. 10.5694/mja13.10920 24999876

[B16] McClaren B. Nisselle A. Prichard Z. Dunlop K. Terrill B. Gaff C. (2018). Mapping Existing Education and Training for the Australian Clinical Genomic Workforce. Australian Genomics, Melbourne: Australian Genomics Workforce & Education Working Group;.

[B17] Miles M. Huberman A. (1994). Qualitative Data Analysis: An expanded sourcebook. 2nd ed. California: Sage.

[B18] Nair A. Chacko J. Pillai S. (2018). A knowledge, attitude, and practices study of pharmacogenomics and its educational needs among doctors in a tertiary care hospital. Natl. J. Physiol. Pharmacy Pharmacol. 0, 1. 10.5455/njppp.2019.9.0722522112018

[B19] Neils-Strunjas J. Guerdjikova A. Christianson C. Nichols L. R. Holbrook D. S. (2004). genetics internet resources for allied health professionals. J. Allied Health 33 (2), 145–149. 15239413

[B20] Peter B. Dougherty M. J. Reed E. K. Edelman E. Hanson K. (2019). Perceived gaps in genetics training among audiologists and speech-language pathologists: lessons from a national survey. Am. J. Speech Lang. Pathol. 28 (2), 408–423. 10.1044/2018_AJSLP-18-0069 31091132

[B21] Pope C. Ziebland S. Mays N. (2000). Qualitative Research in Health Care: Analysing Qualitative Data. Br. Med. J. 320, 114–116. 10.1136/bmj.320.7227.114 10625273 PMC1117368

[B22] Reed E. K. Johansen Taber K. A. Ingram Nissen T. Schott S. Dowling L. O. O’Leary J. C. (2015). What works in genomics education: outcomes of an evidenced-based instructional model for community-based physicians. Genet. Med. 18, 737. 10.1038/gim.2015.144 26583682

[B23] Rubanovich C. K. Cheung C. Mandel J. Bloss C. S. (2018). Physician preparedness for big genomic data: a review of genomic medicine education initiatives in the United States. Hum. Mol. Genet. 27 (R2), R250–R2R8. 10.1093/hmg/ddy170 PMC6061688 29750248

[B24] Skirton H. O’Connor A. Humphreys A. (2012). Nurses’ competence in genetics: a mixed method systematic review. J. Adv. Nurs. 68 (11), 2387–2398. 10.1111/j.1365-2648.2012.06034.x 22607038

[B25] Tonkin E. T. Skirton H. Kirk M. (2018). The first competency based framework in genetics/genomics specifically for midwifery education and practice. Nurse Educ. Pract. 33, 133–140. 10.1016/j.nepr.2018.08.015 30296725

[B26] Wright H. Zhao L. Birks M. Mills J. (2019). Genomic Literacy of Registered Nurses and Midwives in Australia: A Cross-Sectional Survey. J. Nurs. Scholarsh. 51 (1), 40–49. 10.1111/jnu.12440 30367730

[B27] Wynn J. Lewis K. Amendola L. M. Bernhardt B. A. Biswas S. Joshi M. (2018). Clinical providers’ experiences with returning results from genomic sequencing: an interview study. BMC Med. Genomics 11 (1), 45. 10.1186/s12920-018-0360-z 29739461 PMC5941324

[B28] Zant R. S. V. Hwang G. Kerns M. Lindblade K. Toney J. (2015). Genetics education is viewed as important, but not a priority in physical therapist education. J. Physical Ther. Educ. 29 (3), 80. 10.1097/00001416-201529030-00010

